# Supplementary material for: A VPS15-like kinase regulates apicoplast biogenesis and autophagy by promoting PI3P generation in Toxoplasma gondii
Source: PLoS Pathog. 2022 Nov 1;18(11):e1010922. doi: 10.1371/journal.ppat.1010922 (PMC9624415; doi:10.1371/journal.ppat.1010922)
Supplement: S1 Text — (PDF) [file ppat.1010922.s001.pdf]

Supplementary Information

Materials. All the molecular biology reagents were purchased from Sigma-Aldrich/Merck. Oligonucleotides were synthesized from Sigma-Aldrich/Merck, and restriction enzymes were obtained from New England Biolabs, USA. Mouse anti-actin [C4, sc-47778, conjugated to horseradish peroxidase {HRP}] was from Santa Cruz Biotechnology, mouse and rabbit anti-hemagglutinin (anti-HA) were from Roche (Y-11, sc-805) and Cell Signalling Technology (CST) (C29F4), respectively anti-GFP was purchased from Roche, anti-myc from Invitrogen (9E10). The following *Toxoplasma gondii* antibodies were used: anti-GAP45, anti-SAG1, anti-Cpn60 were gifts from Prof. Dominique Soldati-Favre. Horseradish peroxidase-labeled secondary goat anti-rabbit/mouse antibodies (Molecular Probes) were used. Alexa Fluor 488- and Alexa Fluor 594-conjugated goat anti-mouse/rabbit antibodies, Hoechst 33342 were used from Molecular Probes. Anhydrotetracycline hydrochloride, LY294002, paraformaldehyde, Giemsa stain, Methanol, crystal violet, mycophenolic acid, xanthine, phleomycin and pyrimethamine were purchased from Sigma-Aldrich. Aqua Shield-1 (AS1) was bought from Cheminpharma, pGEMT vector was from Promega and TA cloning vector was purchased from Real Biotech, Gibson Assembly cloning kit (E5510S) was purchased from NEB. 2X DyNAmocolor Flash SYBR green quantitative PCR (qPCR) master mix, DMEM, fetal bovine serum, trypsin, antibiotic-antimycotic and Hanks' Balanced Salt Solution (HBSS) were purchased from Thermo Fisher. Most

Toxoplasma-related reagents, which include various vectors and antibodies, were kind gifts from Dominique Soldati-Favre, University of Geneva.
