# Supplementary material for: A VPS15-like kinase regulates apicoplast biogenesis and autophagy by promoting PI3P generation in Toxoplasma gondii
Source: PLoS Pathog. 2022 Nov 1;18(11):e1010922. doi: 10.1371/journal.ppat.1010922 (PMC9624415; doi:10.1371/journal.ppat.1010922)
Supplement: S1 Fig — A. Schematic representation of the strategy used to generate TgVPS15-iKD parasites. A plasmid construct that allowed insertion of transactivator TATi-1 and replacement of TgVPS15 promoter with the 7tet-Op SAG1 (TetO7) inducible promoter was introduced by homologous recombination. In addition, a Myc tag was inserted at the N-terminus of TgVPS15. After transfection, the drug selected parasites were cloned by limiting dilution. B. Genotyping of an independent clone for TgVPS15-iKD parasites. PCR amplification of the endogenous and recombined locus was performed using primers indicated in panel. PCR products of expected size were obtained which confirmed the 5’-and 3’ -integration at the desired locus in TgVPS15-iKD and the endogenous locus (WT) was absent in these parasites but was present in ΔKu80 line. The amplicon obtained by primer set 5F/6R and 7F/8R was sequenced, which confirmed the integration at the expected site. (PDF) [file ppat.1010922.s002.pdf]

A

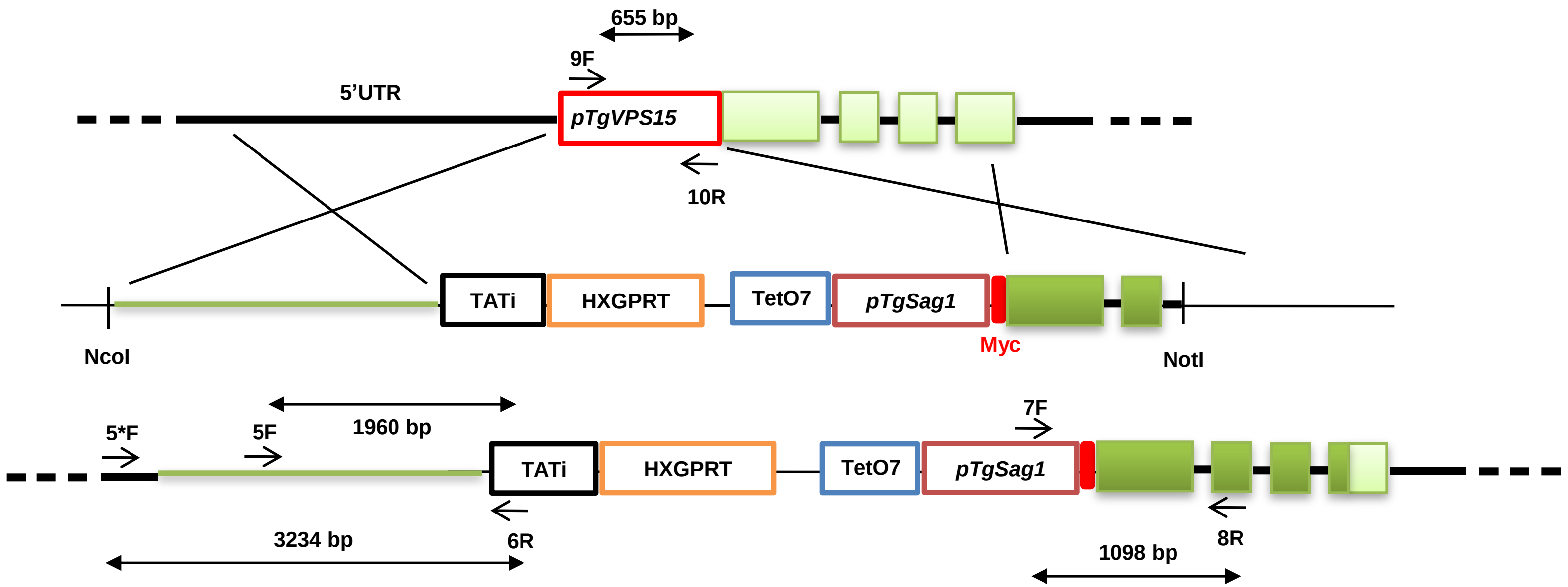

B

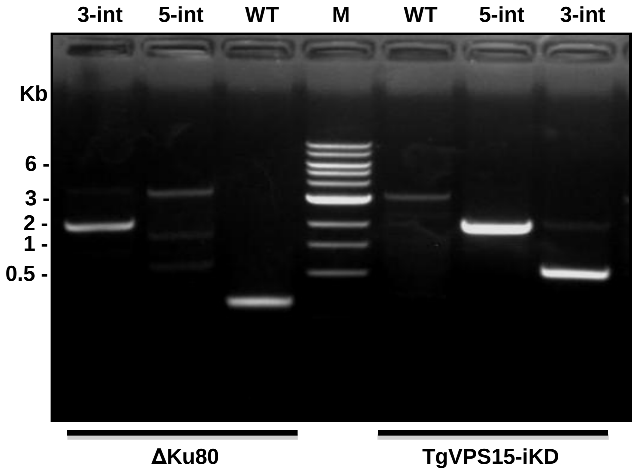

PCR amplicon	PCR primer
WT – 655 bp	9F and 10R
5-integration – 1960 bp	5F and 6R
5'-integration – 3234 bp	5*F and 3R
3-integration – 1098 bp	7F and 8R

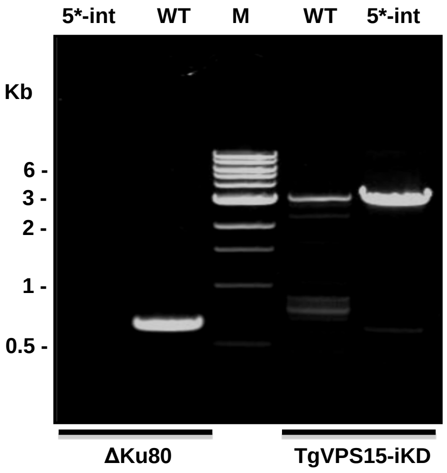

Supp. Fig. S1
