# Supplementary figures and images for: A VPS15-like kinase regulates apicoplast biogenesis and autophagy by promoting PI3P generation in Toxoplasma gondii
Source: PLoS Pathog. 2022 Nov 1;18(11):e1010922. doi: 10.1371/journal.ppat.1010922 (PMC9624415; doi:10.1371/journal.ppat.1010922)

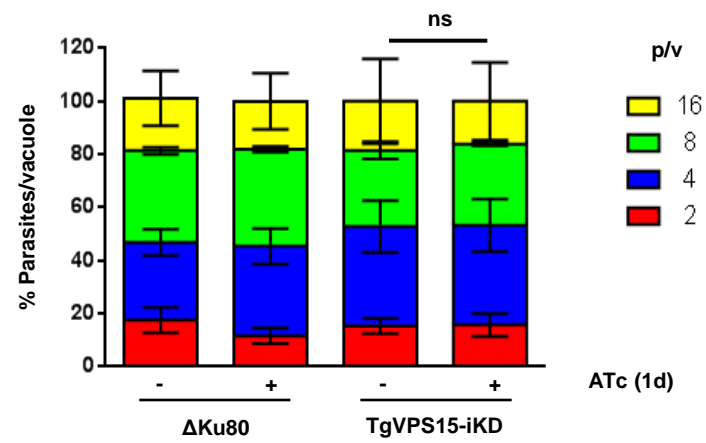

Supplement: S2 Fig — ΔKu80 or TgVPS15-iKD were left untreated (-) or treated (+) with ATc and the number of parasites per vacuole was determined after 24h or 1d. Data represent mean±SE, n = 3 and at least 200 vacuoles were counted for each condition. (PDF) [file ppat.1010922.s003.pdf]

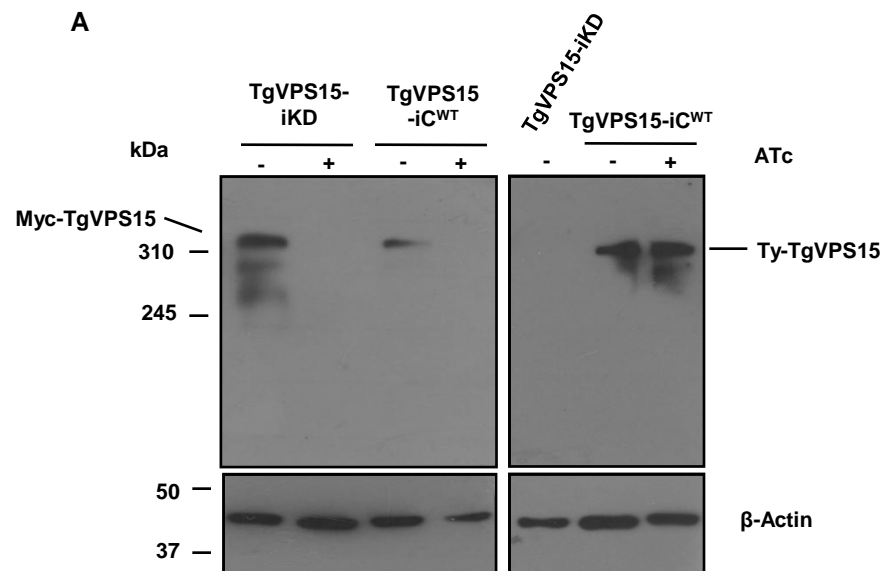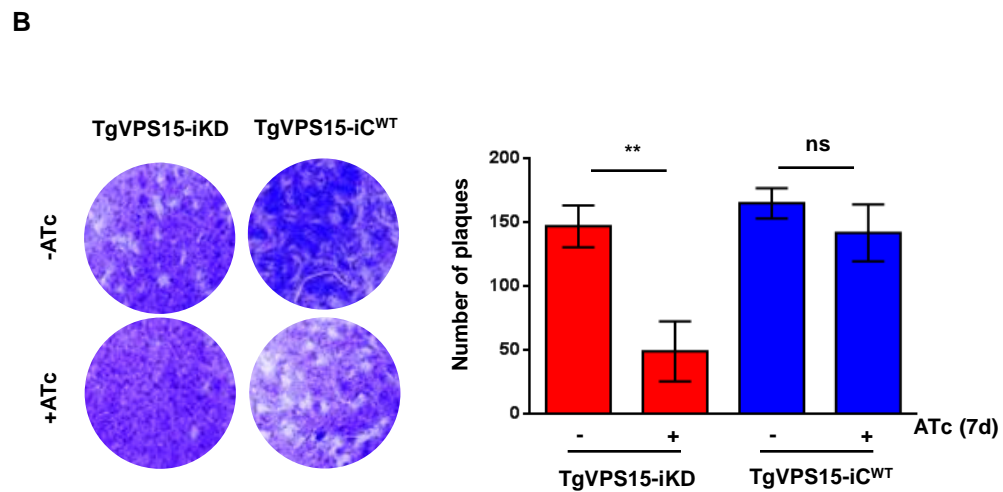

Supplement: S3 Fig — A. TgVPS15-iKD parasites were complemented with a copy of Ty-tagged TgVPS15 (TgVPS15-iCWT). TgVPS15-iKD and TgVPS15-iCWT parasites were treated with ATc for 96h or 4d. Parasite lysate was prepared and used for Western blotting using anti-myc or anti-Ty antibodies. ATc addition depleted Myc-TgVPS15 in both TgVPS15-iKD as well as TgVPS15-iCWT parasites whereas the ectopically expressed Ty-TgVPS15 remained unaffected in TgVPS15-iCWT parasites. B. Plaque assays were performed on TgVPS15-iKD and TgVPS15-iCWT parasites in the presence or absence of ATc as described for Fig 2C and the number of plaques was quantified (Mean±SE, n = 3, **P<0.01, ANOVA ns-not significant). (PDF) [file ppat.1010922.s004.pdf]

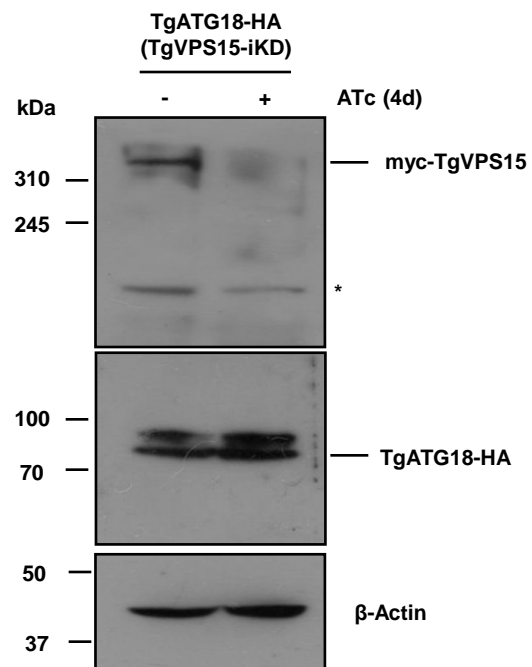

Supplement: S4 Fig — TgVPS15-iKD/GFP-TgATG8/TgATG18-HA parasites were treated with ATc for 4d as described. Western blotting was performed using anti-HA and anti-myc antibodies to detect myc-TgVPS15 and TgATG18-HA. Actin was used as a loading control. (PDF) [file ppat.1010922.s005.pdf]

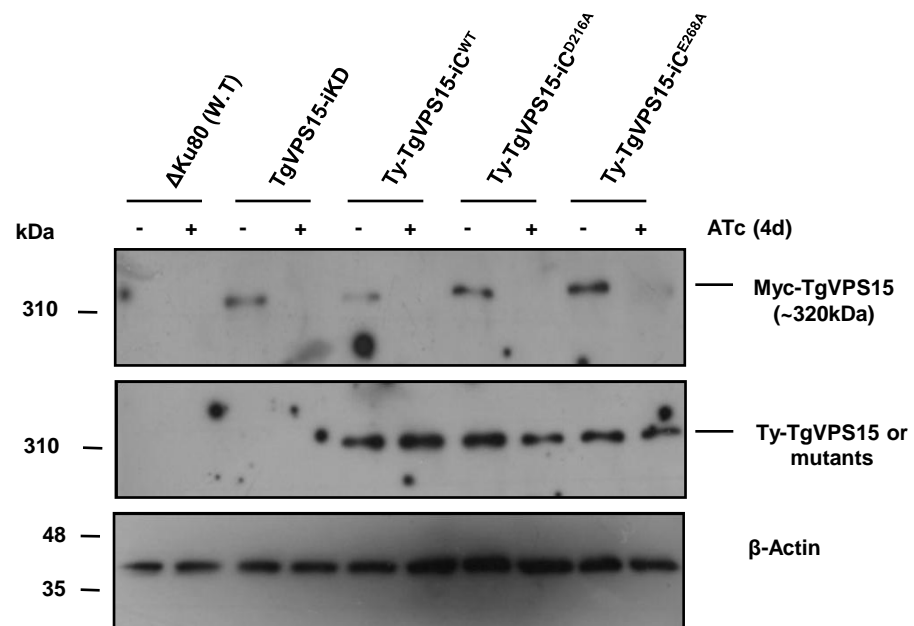

Supplement: S5 Fig — TgVPS15-iKD (Myc tagged TgVPS15) parasites were complemented with a copy of Ty-tagged WT TgVPS15, or its D216A and E268A mutants. The indicated parasite lines were incubated for 96h with ATc and Western blotting was performed using anti-myc or anti-Ty antibodies to detect the inducible Myc-TgVPS15, which was depleted upon ATc treatment, or complemented Ty-TgVPS15 WT/D216A/E268A that remained almost unchanged. Actin was used as a loading control. (PDF) [file ppat.1010922.s006.pdf]

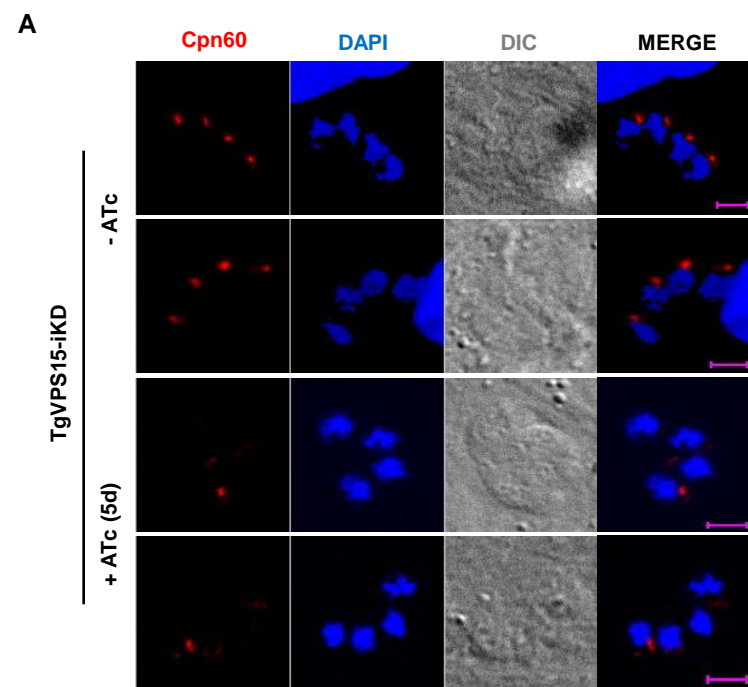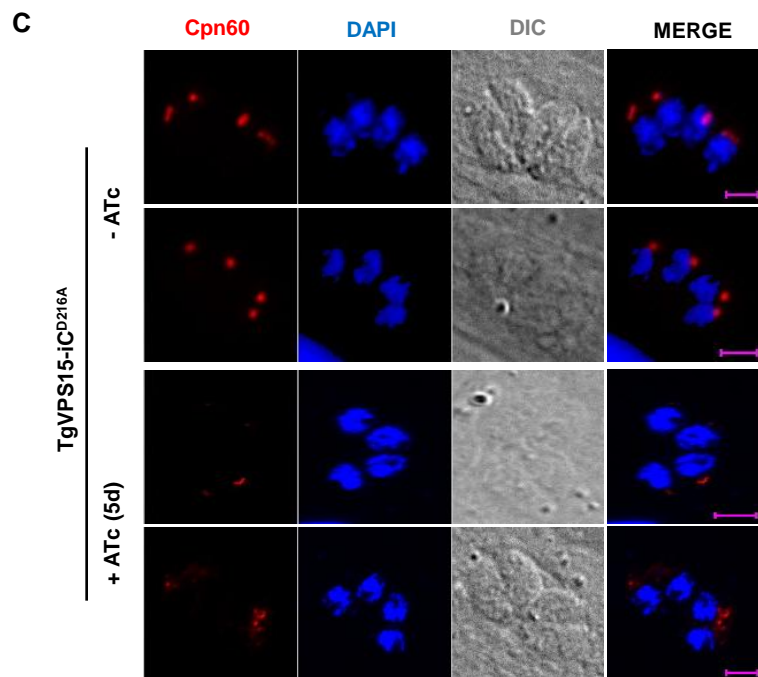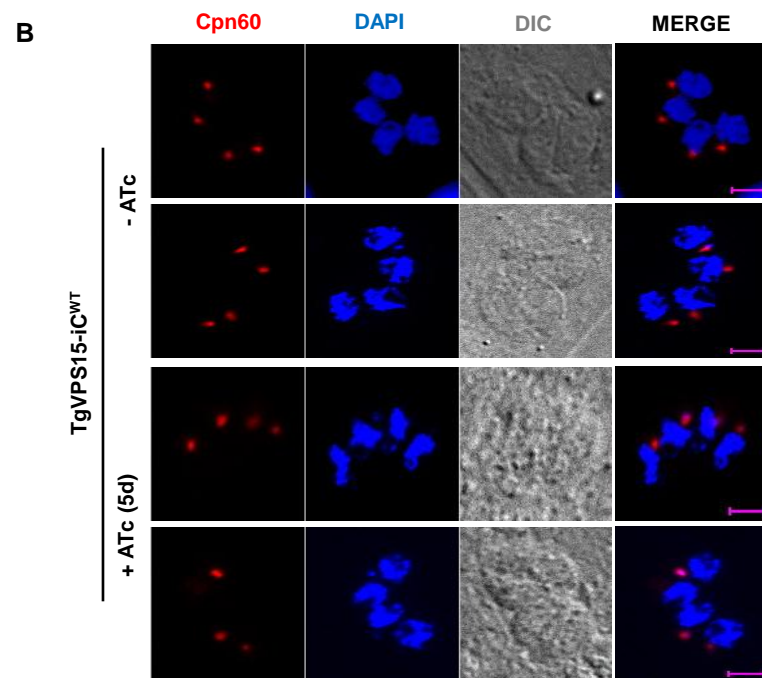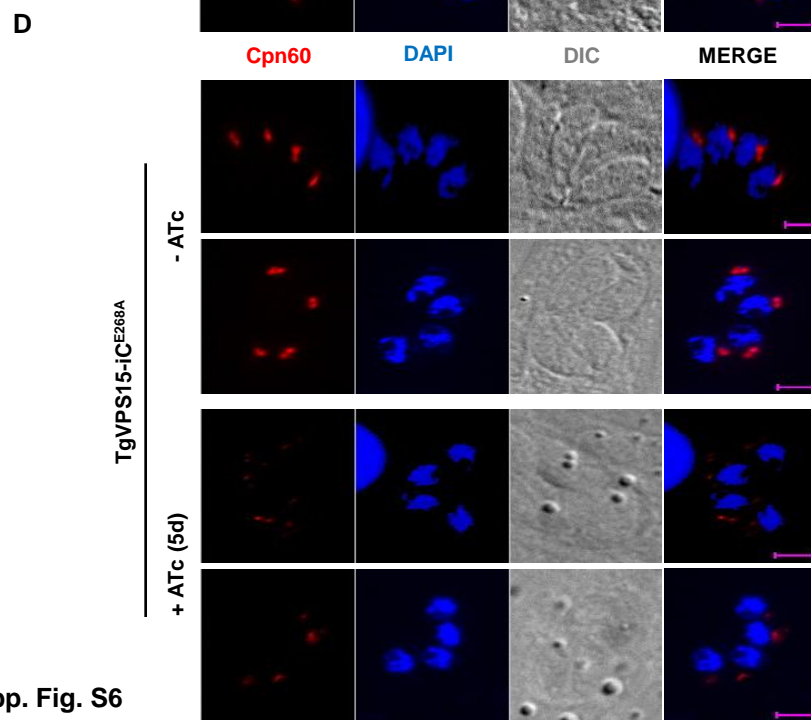

Supp. Fig. S6

Supplement: S6 Fig — TgVPS15-iKD parasites were complemented with a copy of Ty-tagged WT TgVPS15 or its D216A and E268A mutants. The indicated parasite lines were preincubated for 96h with ATc and IFA was performed using anti-Cpn60 antibody after additional 24h. % parasites containing the apicoplast were determined (See Fig 7C). (PDF) [file ppat.1010922.s007.pdf]

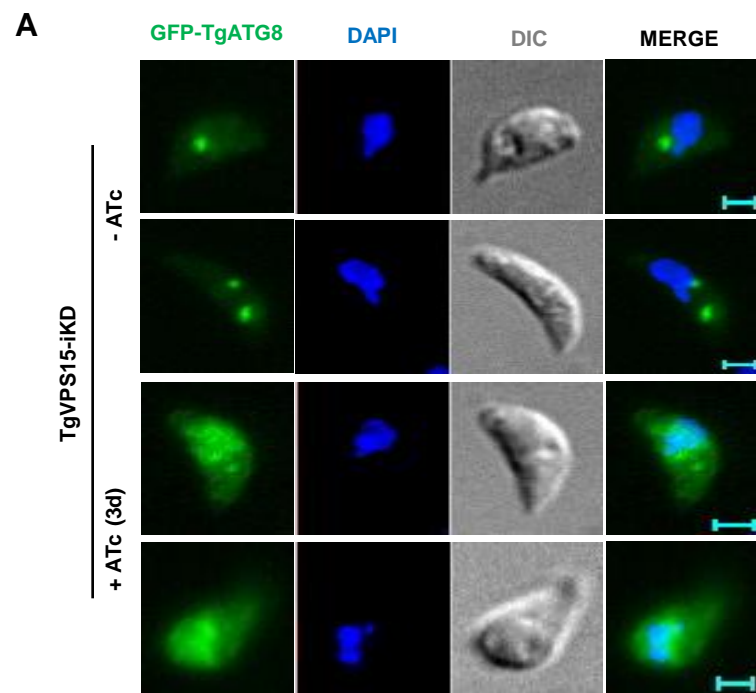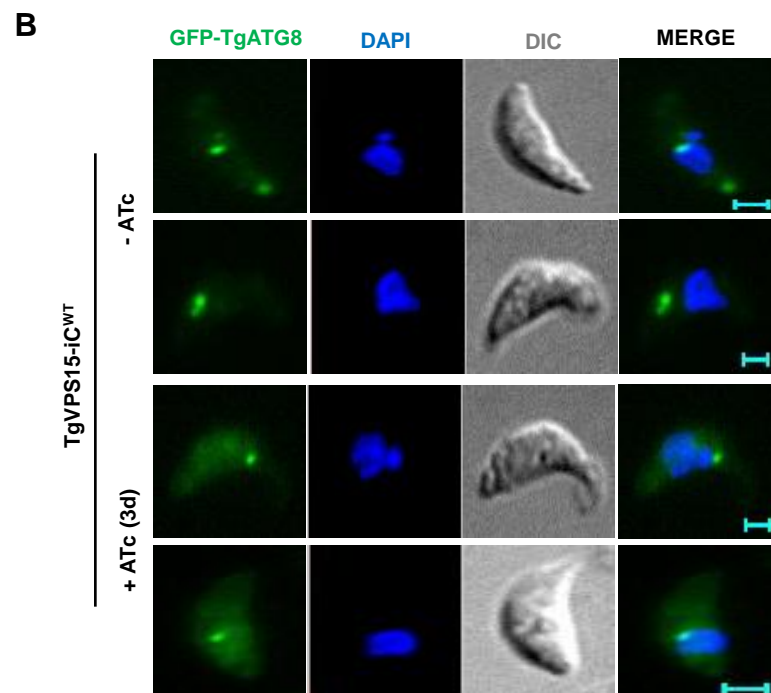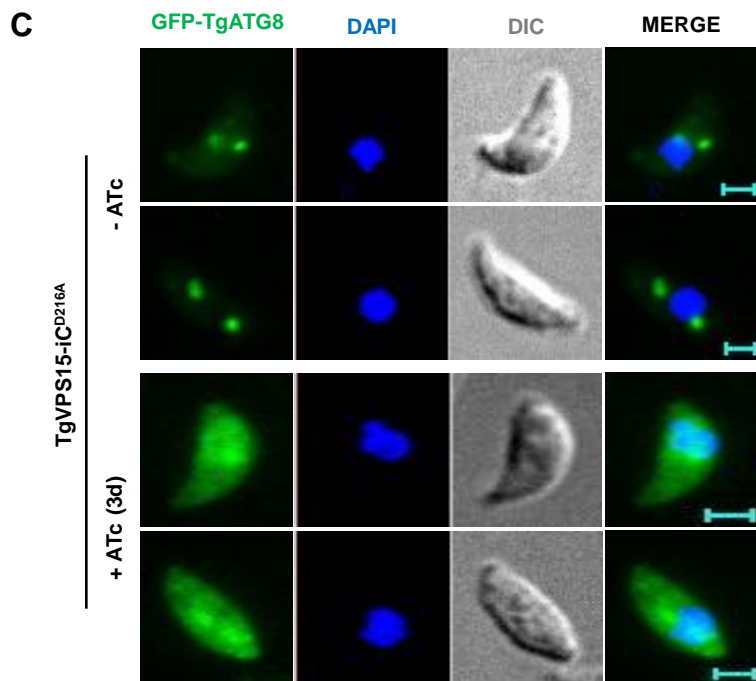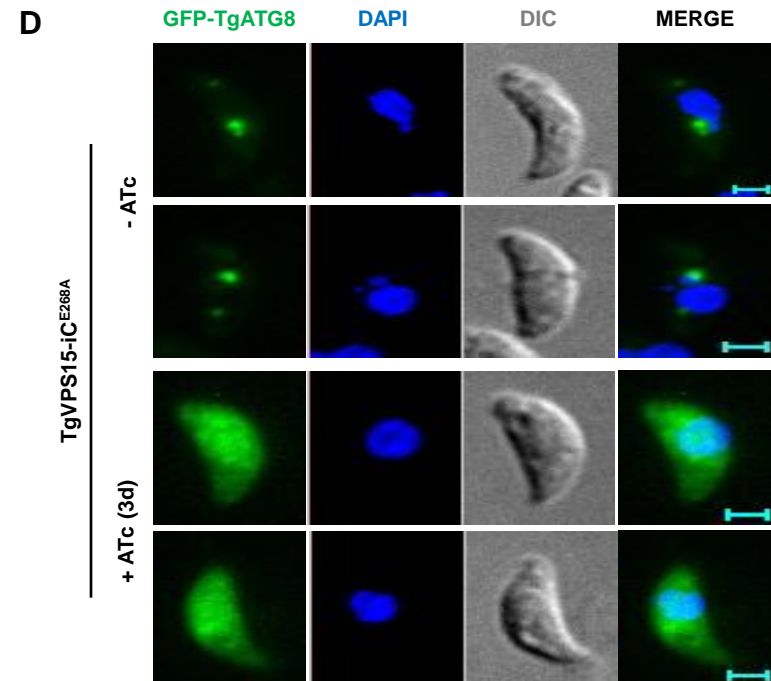

Supplement: S7 Fig — GFP-ATG8 was ectopically expressed in indicated parasite lines and parasites were treated with ATc for 2d and extracellular tachyzoites were cultured in HBSS medium for 8h. Subsequently, parasites were fixed and analyzed by fluorescence microscopy. GFP-ATG8 positive autophagosomes were determined by counting at least 50 parasites and % parasites possessing autophagosomes was determined (see Fig 7D). (PDF) [file ppat.1010922.s008.pdf]
