# Supplementary material for: A VPS15-like kinase regulates apicoplast biogenesis and autophagy by promoting PI3P generation in Toxoplasma gondii
Source: PLoS Pathog. 2022 Nov 1;18(11):e1010922. doi: 10.1371/journal.ppat.1010922 (PMC9624415; doi:10.1371/journal.ppat.1010922)
Supplement: S1 Table — (PDF) [file ppat.1010922.s009.pdf]

Table S1: List of PCR primers

TgVPS15

Purpose	Forward (5'-3')	Reverse (5'-3')
TgVPS15-iKD: 5'-flank Cloning	1 AAAccatggCTCTACAGTGGCAAGGAGGGAAGC	2 AAAggatccAGGAATCCCTAGCCGTCTCACAGC
TgVPS15-iKD: 3' flank cloning	3 (with N-myc) AAAagatctGGCAACACAGTCGCCACGACGAG	4 AAAgcggccgcGAACGAGGAAACGCCGAAGAGGCC
TgVPS15-iKD: 5'-integration genotyping	5 GATTCCGTTGTTTACCGCTTGTGC	6 GTTGATGACTTTGCTCTTGTCCAGG
TgVPS15-iKD: 3'-integration genotyping	7 Ctttccgttcttctcggttgtgtc	8 Gactccaaatgcttgcacagaacg
TgVPS15-iKD: WT locus genotyping	9 AACGCGGAGGTTTCGACTTTAAAAC	10 AGAAGTGAAGGAGACAGACAGGAAG
LIC-DHFR-*TgATG18*-3HA; HA-tagging TgATG18	13 TACTTCCAATCCAATTTAATGCAGCAATCCTGCGCGAATCACCTCGAT	14 TCCTCCACTTCCAATTTTAGCAAACGCTTCCAGACGCTCTGCATGCAGTT
Amplification of DD-GFP from pCT-DD-GFP	15 GGAagatctAAAATGGGAGTGCAGGTGGAAAC	16 atcactttcCCTAGGTTTGTATAGTTCATCCATGC
Amplification of 2xFYVE from p3E-2xFYVE (Hrs)	17 CCTAGGgaaagtgatgccatgttcgctg	18 GCTCgatatcttatgccttcttgttcagc
Cloning of pET28a-TgVPS15-KD	19 GGAcatatgGGTAACACAGTCGCCACG	20 AAgcggccgcATCGGAGACTCCCGGATG
Mutation of TgVPS15 (D216A)	23 CTCGTCCACGGCgccATTAAAGGAGCC	24 GGCTCCTTTAATggcGCCGTGGACGAG
Mutation of TgVPS15 (E268A)	27 CATGTCGCGCCTgcgAGGTTTTTTTCG	28 CGAAAAAAACCTcgcAGGCGCGACATG

pCT-TgVPS15-iC$^{(D216A/E268A)}$		
I. Cloning of Tub8 promoter amplified from pCT	29<br>CCGTTTTCACCATGGGCAAATA<br>TTATACGC	30<br>TTGCAGCCATGGAGAAAACGTCTG
II. Cloning of TgVPS15-D216A/E268A from pET-TgVPS15-KD construct	31<br>CTTGAATTCCCTTTTAGATCTAAAATGCAGGAGGTCCACACGAACCAGGACCCGCTCGATCCTAGGATGGGCAACACAGTC	32<br>GACTGTGTTGCCCATCCTAGGATCGAGCGGGTCCTGGTTCGTGTGGACCTCCTGCATTTTAGATCTAAAAGGGAATTCAAG
NcoI site was used for Gibson cloning	33<br>CAGGACCCGCTCGATCATATGATGGGCAACACAGTC	34<br>GACTGTGTTGCCCATCCTAGGATCGAGCGGGTCCTG

Real Time PCR Primers for apicoplast and nuclear genes:

Oligo	Forward (5’-3’)	Reverse (5’-3’)
TgTufA	TCTATTGCAATGGAAAAAGGTATG	TCAATGGTAGAGCAAAGGACTG
TgUPRT	ACTGCGACGACATACTGGAGAAC	AAGAAAACAAAGCGGAACAACAA
